# 3D Reconstruction of the Clarified Rat Hindbrain Choroid Plexus

**DOI:** 10.3389/fcell.2021.692617

**Published:** 2021-07-29

**Authors:** Paola Perin, Riccardo Rossetti, Carolina Ricci, Daniele Cossellu, Simone Lazzarini, Philipp Bethge, Fabian F. Voigt, Fritjof Helmchen, Laura Batti, Ivana Gantar, Roberto Pizzala

**Affiliations:** ^1^Department of Brain and Behavioral Sciences, University of Pavia, Pavia, Italy; ^2^Department of Molecular Medicine, University of Pavia, Pavia, Italy; ^3^Brain Research Institute, University of Zurich, Zurich, Switzerland; ^4^Neuroscience Center Zurich, Zurich, Switzerland; ^5^Wyss Center for Bio and Neuroengineering, Geneva, Switzerland

**Keywords:** choroid plexus, vascular network, brain ventricle, iDISCO+, tissue clarification

## Abstract

The choroid plexus (CP) acts as a regulated gate between blood and cerebrospinal fluid (CSF). Despite its simple histology (a monostratified cuboidal epithelium overlying a vascularized stroma), this organ has remarkably complex functions several of which involve local interaction with cells located around ventricle walls. Our knowledge of CP structural organization is mainly derived from resin casts, which capture the overall features but only allow reconstruction of the vascular pattern surface, unrelated to the overlying epithelium and only loosely related to ventricular location. Recently, CP single cell atlases are starting to emerge, providing insight on local heterogeneities and interactions. So far, however, few studies have described CP spatial organization at the mesoscale level, because of its fragile nature and deep location within the brain. Here, using an iDISCO-based clearing approach and light-sheet microscopy, we have reconstructed the normal rat hindbrain CP (hCP) macro- and microstructure, using markers for epithelium, arteries, microvasculature, and macrophages, and noted its association with 4th ventricle-related neurovascular structures. The hCP is organized in domains associated to a main vessel (fronds) which carry a variable number of villi; the latter are enclosed by epithelium and may be flat (leaf-like) or rolled up to variable extent. Arteries feeding the hCP emerge from the cerebellar surface, and branch into straight arterioles terminating as small capillary anastomotic networks, which run within a single villus and terminate attaching multiple times to a large tortuous capillary (LTC) which ends into a vein. Venous outflow mostly follows arterial pathways, except for the lateral horizontal segment (LHS) and the caudal sagittal segment. The structure of fronds and villi is related to the microvascular pattern at the hCP surface: when LTCs predominate, leaflike villi are more evident and bulge from the surface; different, corkscrew-like villi are observed in association to arterioles reaching close to the CP surface with spiraling capillaries surrounding them. Both leaf-like and corkscrew-like villi may reach the 4th ventricle floor, making contact points at their tip, where no gap is seen between CP epithelium and ependyma. Contacts usually involve several adjacent villi and may harbor epiplexus macrophages. At the junction between medial (MHS) and lateral (LHS) horizontal segment, arterial supply is connected to the temporal bone subarcuate fossa, and venous outflow drains to a ventral vein which exits through the cochlear nuclei at the Luschka foramen. These vascular connections stabilize the hCP overall structure within the 4th ventricle but make MHS-LHS joint particularly fragile and very easily damaged when removing the brain from the skull. Even in damaged samples, however, CP fronds (or isolated villi) often remain strongly attached to the dorsal cochlear nucleus (DCN) surface; in these fronds, contacts are still present and connecting “bridges” may be seen, suggesting the presence of real molecular contacts rather than mere appositions.

## Introduction

The choroid plexus (CP) acts as a regulated gate between blood and cerebrospinal fluid (CSF). This role involves several functions, such as immune cell trafficking, water and solute transport, and secretion of endogenously produced macromolecules (Neman and Chen, 2016) that are involved in neural metabolism, neuroendocrine signaling, and neurogenic niche regulation (Kaiser and Bryja, 2020). Despite its simple histology (a monostratified cuboid epithelium overlying a connective stroma vascularized by fenestrated capillaries) many aspects of CP functions are still unknown, especially because of its fragility, convoluted shape, and deep location within the brain. So far, the studies observing CP cellular responses *in situ* have shown its interactions with factors in blood and CSF (Cui et al., 2020; Shipley et al., 2020); however, within a single CP, epithelial cells are heterogeneous in their developmental origin (reviewed in Lun M. et al., 2015) and protein expression (Lun M. P. et al., 2015; Dani et al., 2021), especially in relation to intercellular contact (Lobas et al., 2012), and it is not clear whether this heterogeneity translates into spatial differences in CP function. It is known that within ventricles there are CSF currents (Faubel et al., 2016) which would differentially expose the various parts of the ventricular surface to substances released into the CSF (Kreeger et al., 2018), and points of contact of the CP fronds to the ventricular surface have sporadically been described (Terr and Edgerton, 1985). CSF currents and direct contacts would modulate periventricular responses to factors present or released at the apical side of the CP epithelium. For example, macrophages have been observed to cross the CP epithelium upon inflammatory insults (Cui et al., 2020), and CP-derived Otx2 is able to reach neurons influencing their plasticity in the adult (Spatazza et al., 2013). In both cases, CSF currents or proximity could allow the CP to influence a region of the brain without the surrounding tissue being affected.

The recently discovered link between CP geometrical features and human brain pathologies (Lizano et al., 2019) has fueled the interest in a better understanding of the morphology of this somewhat neglected part of the brain. The need for advancing the current knowledge about CP geometry is paradoxically higher in rodents, especially in rat, where standardized anatomical atlases are less complete than for humans (Barrière et al., 2019), despite it being the model of choice for many functional studies of physiology and pathology. So far, few studies have focused on the spatial organization of the organ *in situ*, because of its poor contrast in MRI or CT (Hubert et al., 2019), fragile nature and deep location, which make it difficult to preserve 3D details. Scanning electron microscopy of CP vascular casts from various animals and ventricles have given an overview of vascular network arrangement (Meunier et al., 2013). A recent single-cell and single-nucleus RNA sequencing study has provided a cellular atlas of developing, adult and aging mouse CP across all brain ventricles, and displayed the spatial distribution of specific RNAs and proteins (Dani et al., 2021). However, these studies do not yield enough spatial details to fully reconstruct the connection topology of CP vascular networks and, due to CP isolation from the brain, are unable to assess correlations between vascular patterns, epithelial folding, and ventricular localization.

Using an iDISCO+-based approach and light-sheet microscopy, we have observed in a recent study in the rat hindbrain, the association of CP lateral expansion with auditory system structures, such as cochlear nuclei and endolymphatic sac (Perin et al., 2019). In this work, we employ the same approach to reconstruct the hindbrain CP (hCP) and its components.

## Materials and Methods

Experiments were performed on inbred Wistar rats (*n* = 20: 10 males and 10 females; average age: 116 ± 41 days; mean ± SD). Animals were housed with 12 h/12 h light/dark cycle, food and water provided *ad libitum*; sacrifices were performed during the light phase. This study was carried out in accordance with the recommendations of Act 26/2014, Italian Ministry of Health. The protocol (number 155/2017-PR) was approved by the Italian Ministry of Health and University of Pavia Animal Welfare Office (OPBA). All efforts were made to minimize number of animals used and animal suffering.

### Sample Preparation

For all experiments, animals were anesthetized, sacrificed and fixed as in Perin et al. (2019). Samples were postfixed overnight in 4% PFA and then cryoprotected in 30% sucrose solution until sinking. Although no freezing step was present in the protocol, we found that sucrose cryoprotection greatly enhanced tissue transparency in comparison to non-cryoprotected samples (not shown). In two cases, each half of the hindbrain was imaged separately with different markers, for a total of 22 samples. In nine samples, temporal and basisphenoid bones were left attached to the brain; for better penetration of antibodies, using a rongeur the skull was opened dorsally by removing the parietal and occipital bones, and the tympanic bullae were opened ventrally. Samples including bone were decalcified with buffered 10% EDTA in PBS (pH 7.4, mild shaking, daily changed) until softening of the squamous temporal bone (3–4 weeks at room temperature or 2 weeks at 37°C). Decalcified samples were cleared as in Perin et al. (2019), and immunolabeled using rabbit anti-Iba1 (WAKO, 1:200) for macrophages/microglia, sheep anti-transthyretin (TTR; Abcam n. ab9015, 1:250) or goat anti-Notch-2 (ThermoFisher n. PA547091, 1:50) for CP epithelium, goat anti-rat IgG (ThermoFisher, 1:200) or rabbit anti-collagen IV (ColIV; Abcam n. ab6586, 1:200) for blood vessels, and mouse-anti smooth muscle actin (SMA; Abcam n. ab7817, 1:200) for arteries and veins. Species-matched Alexa-conjugated donkey secondary antibodies (Life Technology) or AttoFluor-conjugated nanobodies (Synaptic Systems) were used at 1:200. Incubations with nanobodies required half the time than with regular antibodies. TO-PRO labeling (2 h, ThermoFisher, 1:2500) was used in some samples to stain cell nuclei at the end of secondary antibody incubation.

### Light-Sheet Imaging

Light-sheet imaging was performed on mesoSPIM microscopes^1^ (Voigt et al., 2019) at the Brain Research Institute, University of Zurich and the Wyss Center for Bio and Neuroengineering in Geneva, Switzerland. Higher resolution scans of two samples were made on a CLARITY-optimized light-sheet microscope (COLM) at the Wyss Center for Bio and Neuroengineering in Geneva, Switzerland.

For mesoSPIM: After staining and clearing, brains were attached to a custom 3D-printed holder, then submerged in a 40 × 40 × 40 mm quartz cuvette (Portmann Instruments) filled with dibenzyl ether (DBE, nd = 1.562) and imaged using a home-built mesoscale single-plane illumination microscope (mesoSPIM). The microscope consists of a dual-sided excitation path using a fiber-coupled multiline laser combiner (405, 488, 515, 561, 594, and 647 nm, Omicron SOLE-6) and a detection path comprising an Olympus MVX-10 zoom macroscope with a 1× objective (Olympus MVPLAPO 1×), a filter wheel (Ludl 96A350), and a scientific CMOS (sCMOS) camera (Hamamatsu Orca Flash 4.0 V3). The excitation paths also contain galvo scanners (GCM-2280-1500, Citizen Chiba) for light-sheet generation and reduction of shadow artifacts due to absorption of the light-sheet. In addition, the beam waist is scanned using electrically tunable lenses (ETL; Optotune EL-16-40-5D-TC-L) synchronized with the rolling shutter of the sCMOS camera. This axially scanned light-sheet mode (ASLM) leads to a uniform axial resolution across the field-of-view (FOV) of 4–10 μm (depending on zoom and wavelength). Image acquisition is controlled using custom software written in Python^2^. Field of view sizes ranged from 16.35 mm at 0.8× (pixel size 8.23 μm) 6.54 mm at 2× (pixel size: 3.3 μm), 3.27 mm at 4× (pixel size: 1.6 μm) to 2.62 mm at 5× (pixel size: 1.28 μm). Z-stacks were acquired at 1, 3, 5, or 8 μm spacing. The laser/filter combinations were: Autofluorescence: 488 nm excitation and a 520/35 bandpass filter (BrightLine HC, AHF); Alexa 555: 561 nm excitation and 561 nm longpass (561LP Edge Basic, AHF); Alexa 633: 647 nm excitation and multiband emission filter (QuadLine Rejectionband ZET405/488/561/640, AHF).

For COLM: Light-sheet imaging was performed using a customized version of the Clarity Optimized Light-sheet Microscope (COLM; Tomer et al., 2014) at the Wyss Center Advanced Light-sheet Imaging Center, Geneva. Briefly, the sample was illuminated by one of the two digitally scanned light sheets, using a 488, 561, and 633 nm wavelength laser. Emitted fluorescence was collected by a 10X XLFLUOR4X N.A. 0.6, filtered (525/50, 609/54, and 680/42 nm Semrock BrightLine HC) and imaged on an Orca-Flash 4.0 LT digital CMOS camera, in rolling shutter mode. A self-adaptive positioning system of the light sheets across z-stacks acquisition ensured optimal image quality over the large z-stack. Images were reconstructed in 3D using the Grid Collection Stitching plugin tool in TeraStitcher (BMC Bioinformatics, Italy). Z-stacks were acquired at 3 μm spacing and pixel size was 0.59 μm

### Image Processing and Analysis

In order to optimize thresholding and segmentation, bubble artifacts were removed manually in Fiji (Schindelin et al., 2012), and striping artifacts were minimized with the VSNR V2 plugin for Fiji (Fehrenbach et al., 2012).

#### Vascular Reconstruction

Vessels were classified as arteries if SMA++ ColIV+, veins if SMA+− ColIV+, with a diameter >50 μm, and an irregular (i.e., not circular) section. Segmentation of arteries and veins (*n* = 7 samples) could be performed with Fiji by Otsu thresholding and binarization of SMA-labeled low-resolution images (pixel size: 3 μm or more). Given that CP arteries and veins are limited in number, branch sparsely and do not taper appreciably, their diameters were measured manually at the CP entering point. Segmented stacks with vessels were skeletonized with the Skeletonize3D FIJI plugin and then the Analyze Skeleton plugin (Arganda-Carreras et al., 2010) was used to extract average branch length, tortuosity (measured as ratio between branch length and endpoint distance) and longest shortest path.

For capillaries, since our non-chain specific anti-Col IV antibody labeled the basal lamina of both blood vessels and CP epithelium (Urabe et al., 2002), leading to the appearance of connecting threads (corresponding to thin stroma between capillaries in a frond), and IgG was also distributed in the stroma, giving similar patterns as Col-IV with connecting threads between vessels (see Figure 8C), capillary segmentation was performed manually by limiting selection to circular or ellipsoid structures (*n* = 4 samples). Capillary tracing was performed on MesoSPIM 5× image stacks or COLM stacks.

**FIGURE 1:**
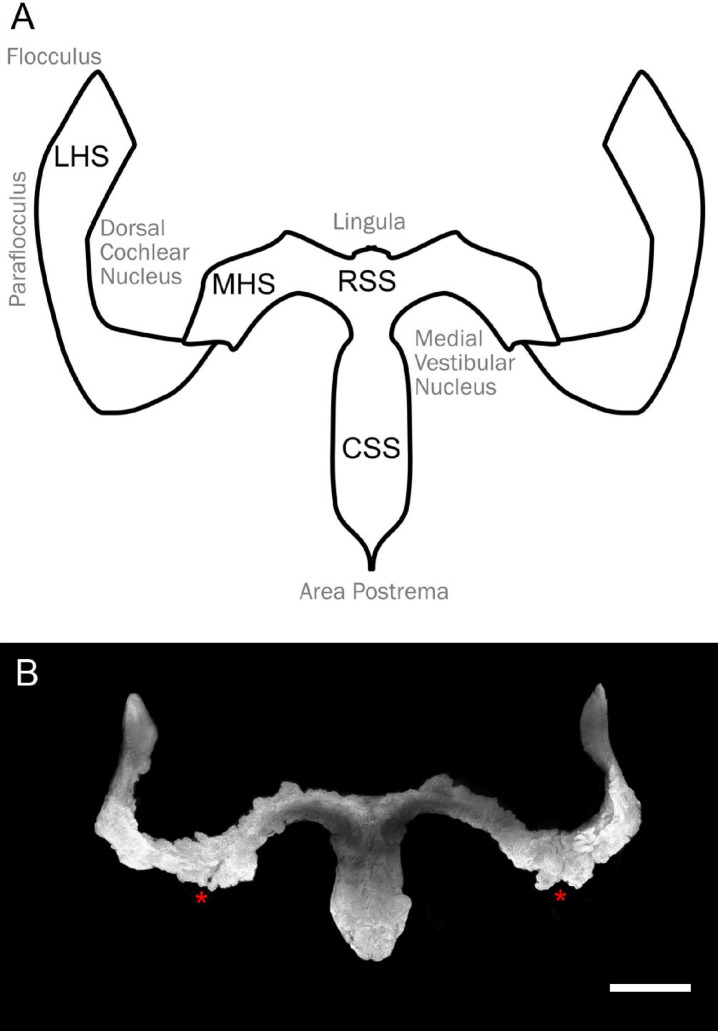
Overall shape of the hindbrain CP. **(A)** Schema of the hindbrain CP with relative hindbrain surrounding areas. RSS, rostral sagittal segment; CSS, caudal sagittal segment; MHS, medial horizontal segment; LHS, lateral horizontal segment. **(B)** Overall CP shape obtained from segmentation of the Notch2 signal Z-stack maximal projection of the light-sheet 3D dataset of a female rat cleared brain. Notice the presence of an incisure between MHS and LHS (asterisks). Scale bar: 1 mm.

**FIGURE 2:**
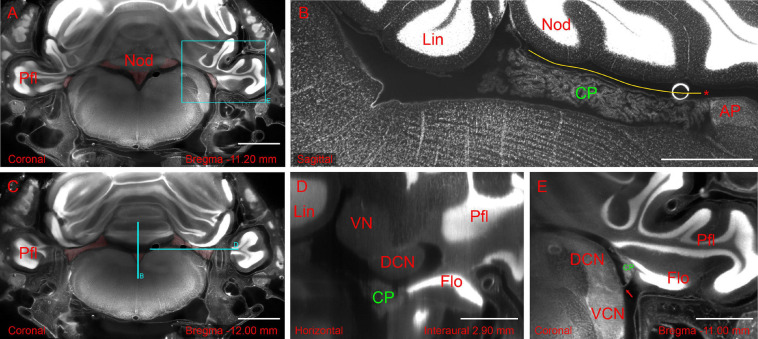
Hindbrain slices from light-sheet 3D dataset of the whole-mount brain-bone preparation showing the extent and association of the CP. **(A)** Coronal slice showing CP attachment (highlighted in red) at the nodulus and paraflocculus. Scale bar: 2 mm. **(B)** Sagittal slice (different animal from **A**) showing the CSS with overlying tela coroidea (yellow line) and attachment to area postrema (asterisk). Scale bar: 1 mm. **(C)** Coronal slice showing the MHS (highlighted in red). Same animal as in **(A)**. Scale bar: 2 mm. **(D)** Horizontal slice detail (reslice of stack in **A,C**) showing the dorsal cochlear nucleus (DCN) and flocculus (FL). Scale bar: 1 mm. **(E)** Coronal slice detail from animal in **(A)** showing the foramen of Luschka (arrow) with the associated CP. Scale bar: 1 mm. All sections were stained with TOPRO for cell nuclei. Autofluorescence signal is also visible. Cyan rectangle on panel **(A)** and cyan lines on panel **(C)** indicate *x*, *y* positions of panels **(B,D,E)**.

**FIGURE 3:**
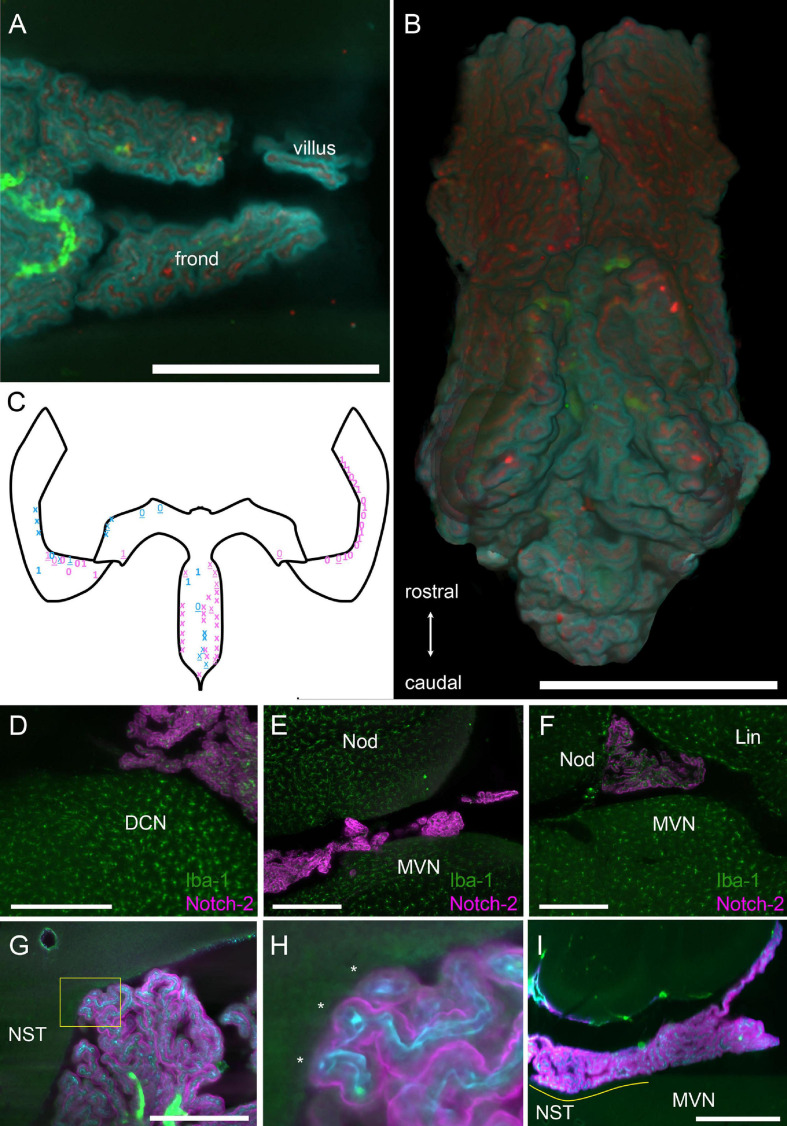
Structure of hCP fronds and villi. **(A)** Example of frond and villus from the CSS. Z-stack maximal projection over 50 μm z-range (5 μm steps, 10 planes). Cyan, Notch-2; red, ColIV; green, autofluorescence and SMA. Scale bar: 1 mm. **(B)** Three-dimensional rendering of the ventral view of the whole SS segmented from the same sample in **(A)**. Scale bar: 1 mm. **(C)** Schema of the CP as in Figure 1A, with indications of location and approximate size of contacts (each symbol indicates a single villus). Blue, males; pink, females. Numbers indicate macrophages at contact site for two animals. Xs indicate two different animals where macrophages were not labeled. Bold symbols indicate tight contacts, underlined symbols indicate loose contacts. In female samples, one was without bone, so only HS was considered, whereas the other was cut in half sagittally, so only HS was considered. In both male samples, right HS was damaged and was not considered. **(D)** Example of foot contacting the DCN. Z-stack maximal projection over 60 μm z-range (2 μm steps). Green, Iba-1 labeling; magenta, Notch-2. **(E)** Foot contacting the MVN. Z-stack maximal projection over 60 μm z-range (2 μm steps). Green, Iba-1 labeling; magenta, Notch-2. **(F)** Foot contacting the lingula. Single 2 μm optical slice. Green, Iba-1 labeling; magenta, Notch-2. Scale bar for **(D–F)**: 400 μm. **(G)** Foot contacting the nucleus of the solitary tract. Single 3 μm optical slice. Green, autofluorescence and SMA; cyan, ColIV; magenta, Notch2 labeling. Scale bar: 500 μm. **(H)** Magnification of COLM image of the contact shown in G (yellow box). Three fronds (asterisks) each containing a capillary are seen contacting the ependyma. **(I)** Single sagittal 5 μm optical plane showing the extent of the largest observed contact area (yellow line). NST, nucleus of the solitary track. Scale bar: 1 mm.

**FIGURE 4:**
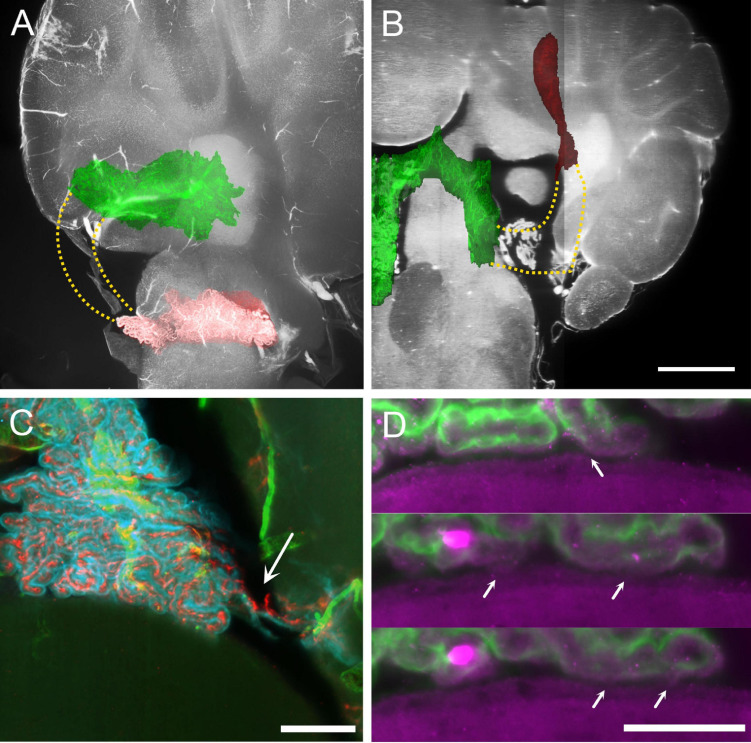
MHS-LHS junction after removal of temporal bone. **(A)** Overlay of plexus vascular segmentation with a substack of unsegmented hindbrain. Red, LHS; green, SS and MHS. The stippled area in yellow corresponds to the location of the missing part of the CP. Sagittal view. **(B)** Similar to **(A)**, resliced to horizontal plane. Scale bar: 1 mm. **(C)** COLM image of a contact region between CP and DCN in a sample where temporal bone has been removed. The plexus appears ripped (arrow) but contact with DCN is not removed. Scale bar: 200 μm. **(D)** Magnification of the CP-DCN contact (same animal as in **C**) showing the indentation marks left by CP villi on DCN ependymal surface. In some positions (arrows) the CP appears connected to the DCN surface. Scale bar: 100 μm.

**FIGURE 5:**
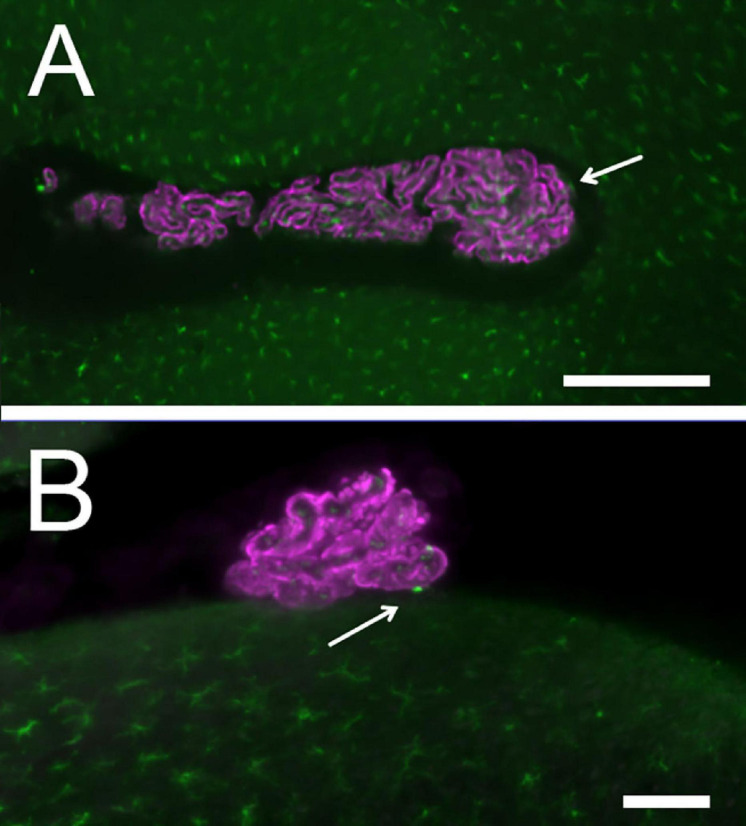
CP macrophage distribution: Epithelium is labeled with Notch2, macrophages with Iba1. **(A)** LHS. Arrows point to epiplexus cells. Scale bar: 400 μm. **(B)** RSS: an epiplexus cell is seen at the contact point with brainstem. Scale bar: 100 μm.

**FIGURE 6:**
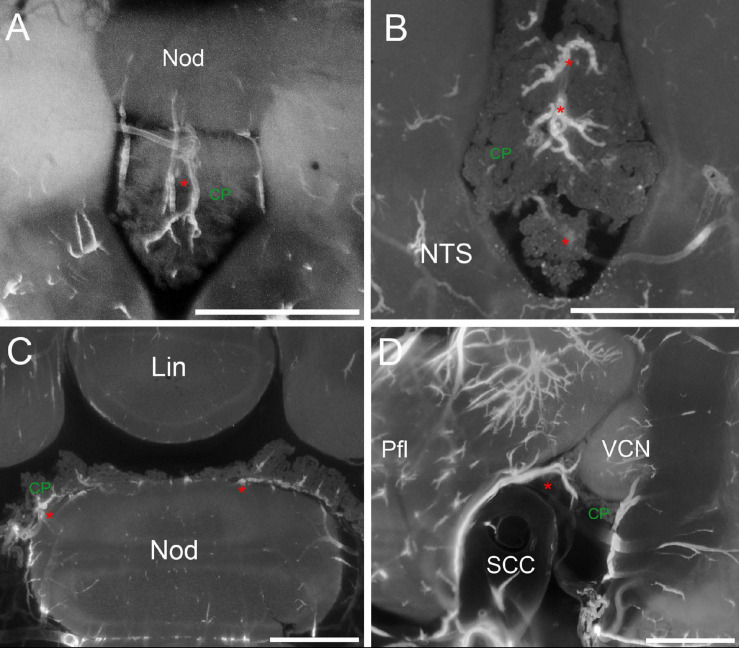
CP arteries. **(A)** Z-stack maximal projection over 90 μm z-range (3 μm steps) showing arteries (red asterisk) penetrating the RSS. **(B)** Z-stack maximal projection over 50 μm z-range (5 μm steps) showing the star-like pattern of arteries entering the CSS. **(C)** Single optical slice showing CP “mounds” around arteries in the MHS and RSS. **(D)** Z-stack maximal projection over 90 μm z-range (3 μm steps) slices showing the artery (red asterisk) penetrating the MHS lateral side from the subarcuate fossa of the paraflocculus. Pfl, paraflocculus; SCC, semicircular canal, VCN, ventral cochlear nucleus. Scale bars: 1 mm. Signal: autofluorescence and SMA.

**FIGURE 7:**
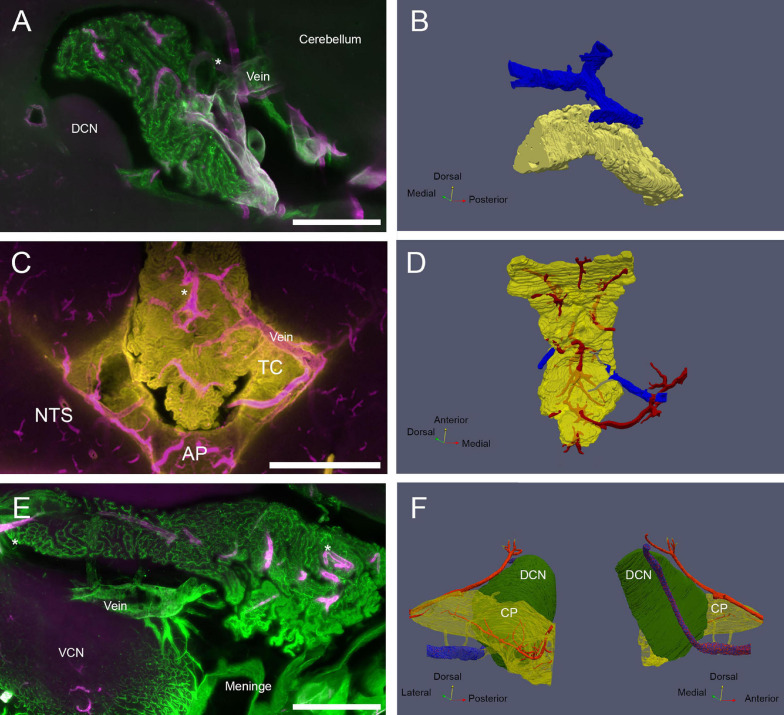
CP veins. **(A)** View of the MHS vascular arrangement. SMA, magenta; ColIV, green. Scale bar: 500 μm. **(B)** Reconstruction of MHS-associated vein. Vein is blue, CP is yellow. **(C)** View of the CSS venous outflow. SMA, magenta; Notch-2, yellow. Note that Notch-2 labels both CP epithelium and tela choroidea (TC). Scale bar: 1 mm. **(D)** Reconstruction of CSS-associated vein. Veins are blue, arteries red, CP yellow (semitransparent). **(E)** View of the LHS vascular arrangement. SMA, magenta; ColIV, green. Scale bar: 500 μm. **(F)** Reconstruction of the vein passing under the DCN and emerging associated with Luschka foramen. Vein is shown in stippled blue. Connections to the CP (shown as semitransparent) are shown in yellow. Arteries (red) feeding the DCN and CP LHS are also shown. Both medial and lateral views are shown for clarity.

**FIGURE 8:**
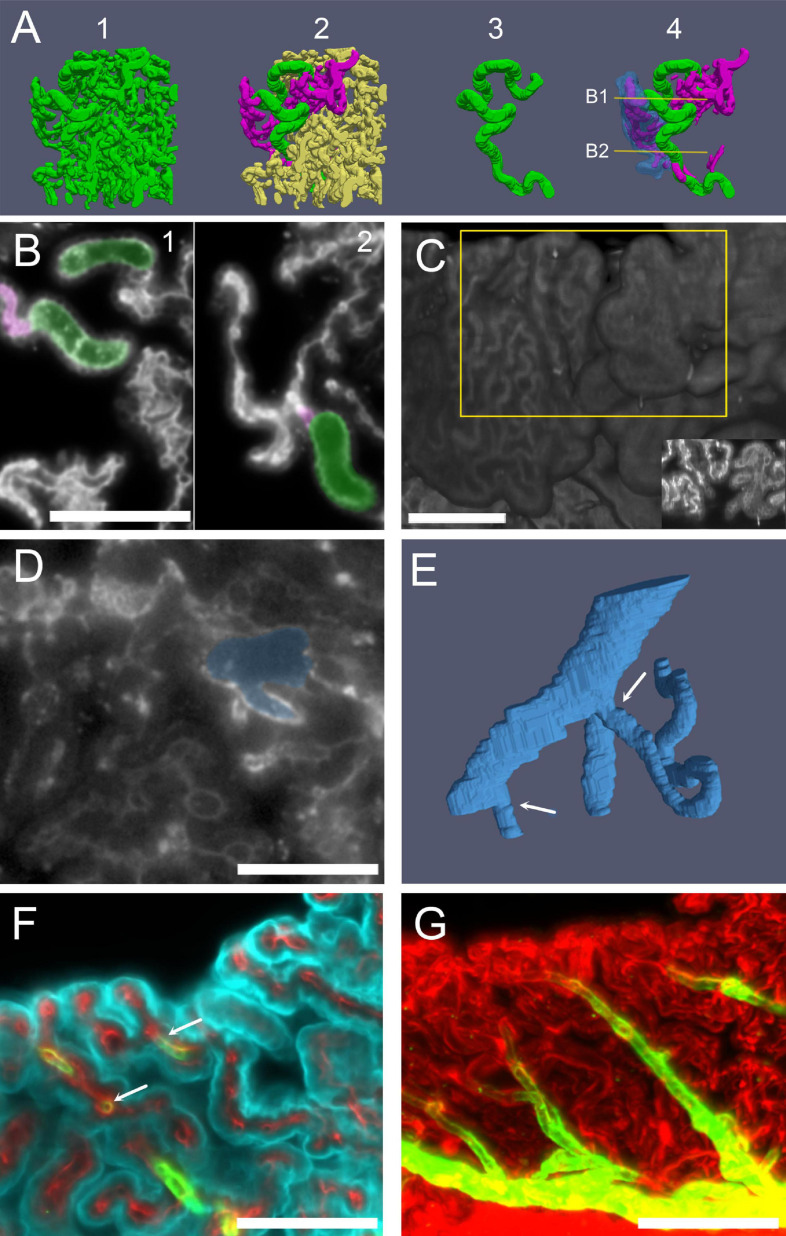
Capillaries. **(A)** Three-dimensional reconstruction of capillaries from a male rat CSS labeled with anti-IgG. Capillaries have been segmented from a cube of CSS CP. 1: All vessels shown together, displaying the full vascular density of the segmented volume. 2: A single large capillary (green) and the small capillaries connected to it (magenta) are evidenced. 3: The large capillary shown in isolation. No branches are present in most large capillaries, which follow a very tortuous path. 4: The large capillary and its connected small capillaries are shown: the epithelium overlying a small capillary anastomotic network (SCAN) is shown in blue (semitransparent). SCANs branch within single villi, which may be folded, making the network appear more complex. Lines and arrows mark the planes and attachment points shown in **(B)**. **(B)** Single optical sections (labeled with anti-IgG) of the stack used for the reconstruction in **(A)**, showing that the SCAN (highlighted magenta) is attached to the same large capillary (highlighted green) at several levels (arrows). Scale bar: 100 μm. **(C)** Three-dimensional reconstruction of the ventral surface of CP CSS labeled with IgG. Villi from two adjacent fronds display only small (left) or only large capillaries (right) on the surface. Scale bar: 200 μm. Inset: Z-stack maximal projection from 10 optical sections from the region highlighted by the yellow box. **(D)** Single optical section (labeled with anti-IgG) showing the junction between a large tortuous capillary and a vein. Scale bar: 100 μm. **(E)** Three-dimensional reconstruction of the capillary-venous junction. Arrows point at the two capillaries entering the vein branches. One of the capillaries is cut short for clarity. **(F)** COLM image of a CP frond showing the arteriole junctions: a straight arteriole (labeled with SMA, green) contacts capillaries (labeled with Col-IV) with the same initial diameter (arrow). Cyan, Notch-2. Since ColIV also labels CP epithelial basal lamina, connections are seen between vessels. **(G)** Z-stack maximal projection of the same sample as **(F)**, showing the whole course of the arteriole (30 μm z-range; 3 μm steps, 10 planes). Red, ColIV; green, SMA. Scale bar: 100 μm.

#### Frond Reconstruction

The choroid plexus epithelium was labeled either with Notch2 or TTR (*n* = 5 samples from four animals). Epithelial outline was segmented with the “snakes” segmentation of ITK-SNAP (Yushkevich et al., 2006) or by Otsu thresholding with Fiji.

Contacts with 4th ventricle parenchyma were annotated following Paxinos atlas (Paxinos and Watson, 2013). Given that iDISCO+ dehydration shrinks tissues, we considered tight contacts to be present when tissues adhered (i.e., no gap was evident) and loose contacts to be present when distance was <10 μm and overall frond shape was complementary to adjacent brain surface. Since not all samples were preserving the entire CP (most samples without bone had damage at the MHS-LHS junction, and samples divided in two sagittally had damage at the SS), number of contacts was compared per section rather than per entire CP.

For macrophages, Iba1 signal (*n* = 8 samples) was thresholded and cells were counted either manually with the ImageJ Cell Counter function or automatedly with the Connected Object function. In samples where an epithelial marker was also present (*n* = 3 samples), stromal and epiplexus populations were counted separately. Macrophages were considered at contact sites when they touched both CP epithelium and ependyma.

#### 3D Reconstruction

Three-dimensional reconstruction was performed with Fiji (Schindelin et al., 2012), ITK-SNAP (Yushkevich et al., 2006), and ParaView (Ahrens et al., 2005; Ayachit, 2015). Briefly, the structures of interest were manually segmented using the Selection Brush tool in FIJI. Segmented stacks were filtered with Gaussian Blur 3D (0.5 pixel for *x* and *y*, 2 pixels for *z*), converted to NRRD and imported in ITK-SNAP, where segmentation was refined using edge-based snakes. Segmented volume was saved as a NIFTI file and surface mesh exported to ParaView for 3D visualization.

### Statistics

Capillary diameters were measured by hand with Fiji using the ColIV or IgG signal on six random-picked high-resolution optical sections; the resulting dataset was fitted with MATLAB function fitgmdist with *k* = 2 in to extract mean sizes of the two capillary populations. Average tortuosity of small and large capillaries was compared using *t*-test assuming unequal variances.

## Results

### Overall CP Shape

As described in other mammals, the rat hCP is composed of four parts on each side (Figure 1). We decided to adopt the human nomenclature (Sharifi et al., 2005), thus dividing the plexus in rostral and caudal sagittal segments (RSS, CSS, located medially) and medial (MHS) and lateral (LHS) horizontal segments. Whereas rostral and caudal SS do not show a clear division between them, clear boundaries are instead observed between RSS and MHS and between MHS and LHS.

The hCP is dorsally attached to the cerebellum. In the rat, the RSS emerges from the ventral side of the cerebellar nodulus and the MHS from its lateral side (Figure 2A). The RSS emerges as a bilateral expansion on the ventral side of the nodulus, but the two sides merge while going posteriorly, and detach from the nodulus because of the interposition of the tela choroidea (Figure 2B). At the caudal end of the ventricle, the CSS is anchored to the area postrema (Figure 2B). The MHS nodulus expansions extends in the latero-posterior direction showing a partial overlap with a different expansion coming from the paraflocculus (Figure 2C), which reaches the opening of the subarcuate fossa and curves anteriorly surrounding the dorsal cochlear nucleus (Figure 2D), emerging from the Luschka foramen at the junction between the dorsal and ventral cochlear nucleus, and ending in association to the ventromedial side of the flocculus (Figure 2E). The convolute structure of the hCP follows that of the cerebellum, with the HS following the lateral expansion of the flocculonodular lobe and the SS extending backward. The overall shape of CP and its association with brain structures are also shown in Supplementary Video 1.

### Fronds

The epithelial surface of the hCP is folded in a complex way. We will call “frond” a 3D region of the CP related to a parent vessel, which appears separable from the adjacent CP when labeled with an epithelial marker, and “villus” a (usually) sheet-like projection containing two layers of epithelial cells and the related capillaries (Figure 3A). Frond geometry changes depending on CP region, and flat, compact regions alternate with looser configurations, with thin, convoluted fronds and villi (Figure 3B). Frond exact shape and position are poorly conserved among individuals, but they may form physical contact points with several structures located at the ventricular surface (Figure 3C); in particular, the dorsal cochlear nucleus (Figure 3D), medial vestibular nucleus/prepositus hypoglossi (Figure 3E) cerebellar lingula (Figure 3F) and nucleus of the solitary tract (Figures 3G,H). Sporadic frond contacts are also seen around the arterial entry points at the cerebellar surface (not shown). At these contact points, villi touch the brain surface. Although isolated villi may make contact points, at most sites adjacent or close villi form multiple contact points (Figures 3C,H), and the resulting structure may follow the brain surface for 100–200 μm or more: the largest contact seen (Figure 3I) covered an area of 2 × 10^5^ μm^2^ overall. Although CP epithelium was labeled and observed at high resolution in four animals only (two males and two females), it is interesting to note, as a preliminary observation, that epithelial contacts measured in females appeared more numerous than in males (27 ± 7 per segment, vs 11 ± 2) and tighter (loose contacts were 13 ± 0.2% in females vs 45 ± 4% in males).

Because of its loose configuration and attachment points with surrounding structures, the junction between MHS and LHS appears to be the most fragile part of the plexus, and in preparations where the brain has been taken out of the skull it may be frayed or entirely missing (Figures 4A,B). This region is also often damaged in available published atlases. In samples where the bone was removed preserving the CP MHS-LHS junction, contacts were still found in this region, often displaying ripped vascular connections (Figure 4C); interestingly, at high resolution, ependyma displayed surface indents corresponding to overlying villi, and points where small connecting bridges were seen between ependyma and CP villi (Figure 4D), suggesting the presence of molecular bonds (rather than just loose apposition) between the CP epithelium and ependyma.

In samples where the epithelium was co-labeled with Iba-1 (*n* = 2, one male and one female), the large majority (92 ± 2%, *n* = 1914) of macrophages were stromal and spaced in a regular way (Figure 5); epiplexus macrophages were observed in all positions around the epithelium, including contacts with the ventricular surface, located between the CP foot and the ependyma (Figure 5B). Half of contact points (*n* = 36) displayed epiplexus macrophages, usually a single one (see Figure 3C).

### Vascular Network

The CP artery supply (Figure 6) is mainly derived from the AICA (a minor contribution from SCA was seen in one rat). At CP attachment points to the cerebellum, arteries running on the cerebellar surface periodically bifurcate forming thin and scarcely branching arteries which enter the CP. At entry points (which are only broadly conserved among animals, Supplementary Figure 1A) a single artery, a small cluster, or a branch of a parent artery coasting the CP may be found. The SS arteries run dorsoventrally from the nodulus, with a straight course in the RSS (Figure 6A), and a more branched distribution in the CSS (Figure 6B).

Arteries for the MHS emerge periodically from the flocculonodular lobe of the cerebellum, starting from the nodulus side and running at the dorsal side or in the center of the MHS core (Figure 6C). Arteries deriving from the paraflocculus are fed by the AICA through an artery loop within the subarcuate fossa, which exits the fossa to reach the CP (Figure 6D). A final arterial entry point is seen at the LHS anterior tip (see Figure 6A or later in Figure 7F), which is independent from the parafloccular loop. CP arteries are usually quite thin (37.7 ± 13.6 μm at CP entry point, *n* = 77), straight (tortuosity index = 0.81 ± 0.11), and branch with low complexity (on average, the maximal branching order from CP limit to SMA disappearance is 1.6 ± 0.5) after entering the CP.

Venous drainage (Figure 7) is carried by vessels which are much larger (average diameter: 106 ± 39 μm, *n* = 18) but less numerous (Supplementary Figure 1B) than arteries. The RSS and MHS drain dorsally into veins running to the cerebellum (Figures 7A,B). The CSS drains laterally into a vein passing through the solitary tract (Figures 7C,D), which enters the plexus through the tela choroidea and joins dorsal arterial supply upon entry. The LHS is instead drained by ventral veins (Figures 7E,F) which converge into a larger vein running under the DCN and exiting the brain parenchyma at the foramen of Luschka, where it associates with the meningeal rim. The same vein receives blood from the cochlear nuclei: directly from the DCN and indirectly, through short tributaries, from the VCN.

Differently from arteries and veins, CP microcirculation (Figure 8) displays a peculiar organization. Measured capillary sizes were fit with two gaussian-distributed populations: large (average diameter 31.5 ± 4 μm) and small (average diameter: 15.6 ± 3 μm). The spatial pattern of the two capillary types is very different (Figure 8). Large capillaries display high tortuosity (tortuosity index: 0.58 ± 0.18, *n* = 10), and little branching into other large capillaries (Figure 8A). Small capillaries are significantly less tortuous (tortuosity index: 0.83 ± 0.09, *n* = 255, *p* = 0.01), and connect to large tortuous capillaries (LTCs) forming bidimensional anastomotic networks (SCANs, small capillary anastomotic networks, containing interconnected segments with a mean length of 60 ± 30 μm) (Figures 8A,B) which fill single villi (Figure 8A and Supplementary Videos 2A–D). A quantification of the two populations’ proportions and positions relative to the edge of the villi of large and small capillaries in a single frond will require segmentation of the whole vascular network at high resolution. However, it is well apparent that the distribution of small and large capillaries is not homogeneous even in adjacent fronds (Figure 8C), and therefore extreme caution must be exerted when measuring effects of a treatment on CP capillary diameter. As discussed later, part of the heterogeneity is regionalized within CP segments.

Although we did not, in the present work, reconstruct the whole artery-to-vein vascular network, in high-resolution samples we could observe and reconstruct junctions among all vascular elements. Large capillaries were seen connecting to veins (Figure 8D), and appeared large and tortuous until the end, draining into the principal vein or one of its short branches (Figure 8E). Therefore, these large capillaries may be considered venules. Arterioles, recognized by their SMA+ muscle layer, were straight, thin, and gave origin to small capillaries (Figure 8F), usually with a short SMA+ segment emerging at an angle (Figure 8G).

**FIGURE 9:**
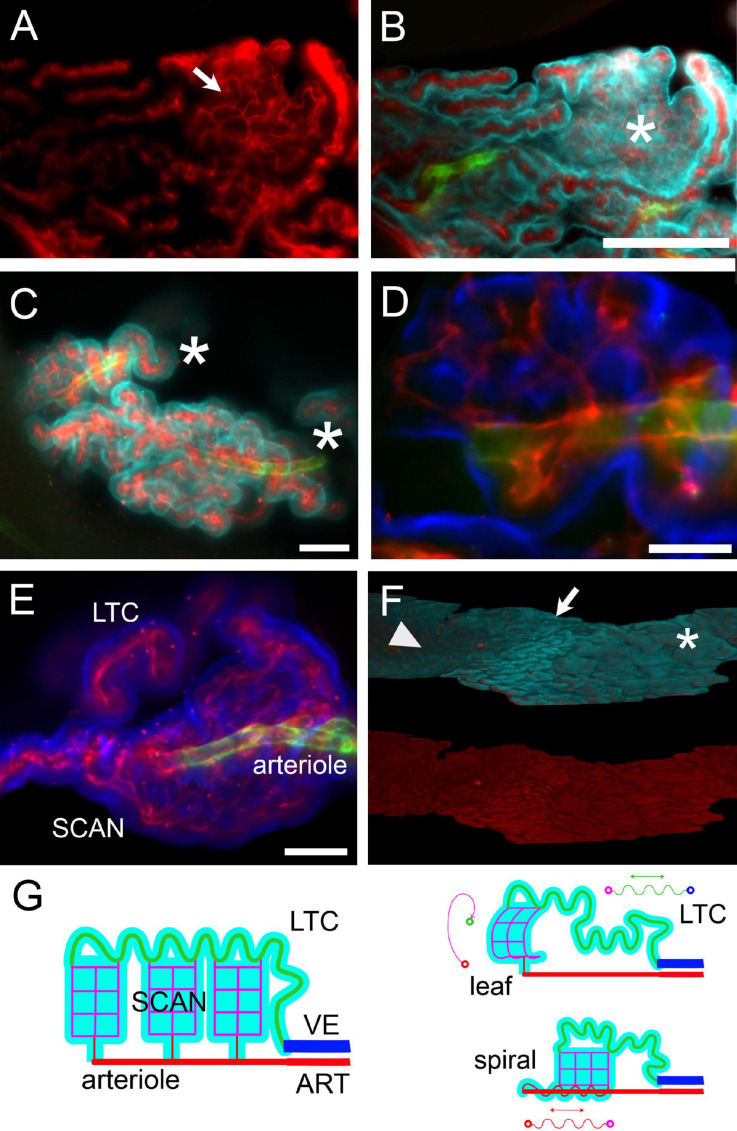
Microvascular network and frond structure. **(A)** SCAN within a single villus in the loose part of LHS of a female rat. The anastomotic pattern of the small capillary network (arrow) is evident. Collagen IV labeling. **(B)** Same section as in **(A)**, additionally labeled for CP epithelium (Notch2, cyan) and arterioles (SMA, green). One flat villus is *en face* in this section (asterisk), and its whole lateral surface is visible. The distribution of large capillaries at the periphery and small capillary (SCAN) in the center is evident. This SCAN attaches to its parent arteriole following a loose spiral course (see Supplementary Video 6). Scale bar: 200 μm. **(C)** Terminal region of corkscrew-like villi from the loose part of CSS. Arterioles are seen in the middle, associated with spiraling capillaries. Asterisks mark the direction of the parent artery. Green, SMA; red, ColIV; cyan, Notch-2. Scale bar: 100 μm. **(D)** Single optical slice with a magnification of a portion of the top villus in **(C)**, showing that the arteriole is associated with capillaries running within the same villus. Scale bar: 50 μm. Green, SMA; red, ColIV; blue, Notch-2. **(E)** Z-stack maximal projection from 50 optical sections (3 μm steps) displaying a SCAN associated with its arteriole and LTC. Scale bar: 100 μm. **(F)** Three-dimensional reconstruction of the LHS surface of a female rat facing the DCN. Top: Notch-2 (cyan) + ColIV (red) + SMA (green) labeling. Bottom: colIV labeling (red). Notice the passage from leaflike villi (asterisk) to digitiform villi (arrow) to flat surface (arrowhead) passing from the caudal to the rostral part of LHS. Scale bar: 500 μm. **(G)** Schema of hCP vascular network. Arteries (ART) divide into arterioles, which give rise to SCANs (magenta) through divergent connections. At their other end, SCANs contact LTCs (green) which course back to reach veins (VE). Since LTCs are very long, a single capillary may fill an entire frond, and SCANs are attached *via* multiple outlets to a single LTC. Probably due to growth with spatial constraints, both SCANs and LTCs may display a tortuous pathway; differential growth of SCANs and LTCs (and possibly pre-SCAN vessels) may produce all microvascular patterns observed.

Microvascular patterns were associated with frond structure (Figure 9). When both large and small capillaries are found within a single leaflike villus (Figures 9A,B), the large capillary usually followed the free margin. However, connections from the venular end of the villus to the arteriolar end often followed a global spiral pathway (see Supplementary Video 3). In the loose part of CSS (shown at low-res in Figure 3B), single capillaries could instead be seen spiraling in association with their feeder arterioles (Figure 9D; see also Figure 8B1); the spiral was covered by epithelium with little or no interposing stroma (Figure 9E). Leaflike villi covering SCANs were also present in the loose CSS, both associated to arterioles (Figure 9F) and venules (e.g., the one in Figure 8A); in these cases, villi were usually folded around the main vessel. In the loose part of LHS, capillaries defined flat leaflike villi, whereas in compact fronds, no protruding structure was observed on the free surface, which displayed ordered rows of capillaries (Figures 9A,F, see also Figure 8C). A tentative schematic reconstruction of the CP vascular network is depicted in Figure 9G.

## Discussion

The CP performs a multitude of important functions including CSF production, development, and maintenance of the periventricular neurogenic zones, neuroimmune communication, and delivery of micronutrients, growth factors, hormones and neurotrophins to the brain *via* the CSF, either from systemic blood through the leaky microvasculature or from the CP epithelium (Ghersi-Egea et al., 2018; Kaiser and Bryja, 2020) or mesenchymal components (Dani et al., 2021). The association between CP vascular, stromal, and epithelial components appears essential for CP functioning, also due to the presence of local signaling between epithelial cells, mesenchymal cells, and blood vessels. Given that molecular heterogeneity in the roof plate precursors of hCP appears to persist in the adult, with the segregation of cell lineages that do not intermingle (reviewed in Lun M. et al., 2015), and that Shh-dependent production of CP epithelial cells and blood vessels is also regionalized (Huang et al., 2009), it appears likely that some spatial variations of epithelial features would correlate with those of vascular network. A recent work characterizing RNA expression at cell level and mapping the expression of selected RNAs and proteins has shown that several genes are expressed along a rostrocaudal gradient. Interestingly, Shh appears more expressed in the rostral part, and may be involved in the heterogeneity of frond compactness we observed in the present work, which also follows a rostrocaudal distribution.

In recent years, several vascular atlases of rodent brains (Quintana et al., 2019; Kirst et al., 2020; Todorov et al., 2020) have been produced at capillary detail. However, the most detailed atlases focus on brain parenchyma, whereas CP is usually overlooked or just mentioned in passing. The CP capillary bed is however very different from those of the brain parenchyma (Cserr, 1971; Parab et al., 2021), and in the hCP these differences are carried to the extreme (Gomez and Gordon-Potts, 1981; Weiger et al., 1986; Scala et al., 1994). Moreover, the rodent hCP presents as an additional challenge its association with the paraflocculus in its most lateral region, which makes it extremely easy to damage when removing the brain from the skull. This work sets a primer for further morphological analysis of the hCP on clarified rat preparations.

In the rat, lateral CP vascular casts show parallel arteries feeding a flat, highly anastomosed capillary network draining into parallel veins that follow artery courses (Sangiorgi et al., 2003), and in mouse immunolabeling shows tight straight arteries expressing claudin-5 and vWF+ veins following arteries and also coasting the CP free edge (Dani et al., 2021). On the other hand, in the rat hCP venules were observed to be tortuous, but their connections to capillaries were not explored (Sun and Hashimoto, 1991).

Our reconstruction of the vascular architecture suggests that in the rat hCP there are two microvascular compartments, namely, SCANs and LTCs. The regularity of arteriolar sprouting could suggest the presence of SCAN “modules” that can be interposed between arterioles and LTCs (which are venular in position). Although SCANs contain vessels of smaller diameter than LTCs, anastomoses between them would make parallel channels, increasing the total area for blood flow. Simulations employing the actual geometry of the CP vascular network will be needed to understand blood flow features in SCANs and LTCs. Several questions arise from the present data: 1-is the permeability similar in SCANs and LTCs? Single-cell expression in the mouse did not evidence distinct endothelial populations. Moreover, our IgG data suggest that both small and large capillaries are permeable to proteins, since both displayed outer IgG rings. Consistently, arterioles [which have been found to display tight walls (Dani et al., 2021)] were clearly observed in SMA-labeled samples but no straight vessels with a diameter similar to SMA+ arterioles were seen in IgG-labeled samples. 2- are SCANs dynamically regulated as modules? SCAN confinement within single villi could allow local interactions between endothelium, stromal components, and epithelium, such as what has been evidenced in Dani et al. (2021). 3- is there any feedback between LTCs and feeder arterioles? Our images evidenced the presence of SMA+ sphincters at SCAN sprouting from arterioles, but we could not clearly identify arteriovenous shunts. However, since CSF production is highly regulated, the dynamic diversion of flow to large capillaries *via* arteriolar vasoconstriction, and the modular organization of small capillaries (which may be optimized in number and size *via* vascular remodeling) suggest interesting hypotheses for functional regulation.

During development, both angiogenesis and epitheliogenesis are regulated by Shh in the hCP but not telencephalic CPs (Nielsen and Dymecki, 2010). In the adult animal, the CP epithelium has been found to be postmitotic (Huang et al., 2009) or dividing at a much slower rate than during development (Dani et al., 2021), and therefore vascular growth in a fixed-volume villus or frond would be expected to induce tortuosity. Although the action of Shh appears restricted to development in normal animals (Nielsen and Dymecki, 2010), it is likely that physiological or pathological stimuli activate angiogenesis in the adult: for instance, VEGF is released from CP epithelial cells and affects both CP endothelium and ependyma (Maharaj et al., 2008), and redundant VEGF signaling pathways, both cell-autonomous and non-autonomous, are involved in the formation of fenestrated endothelium in zebrafish CP (Parab et al., 2021). Dynamical vascular remodeling would be particularly expected in the hCP, since a differential transcriptome analysis (Lun M. P. et al., 2015) displayed that angiogenesis-related genes are more highly expressed in it than in telencephalic CP. Moreover, the localization of dividing cells mostly close to CP attachment (Dani et al., 2021), where veins are found, could suggest that LTC tortuosity comes from vascular growth at the venous end, pushing the vessel toward CP distal villi.

In regards to rat hCP macroanatomy, the observed peculiar arrangements of arteries and veins most likely reflect development of the cerebellum and related structures. In fact, the rodent paraflocculus extends laterally, reorients during development, and ends surrounded by temporal bone (Panezai et al., 2020); on the other hand, the dorsal cochlear nucleus is a paracerebellar nucleus that shares a similar (though not identical) developmental origin with the cerebellum (Farago et al., 2006), and therefore association of CP-related vasculature to cochlear nuclei may reflect rhombic lip rearrangements during development.

Since the overall CP structure is stabilized by the macrovascular connections, our finding that villi may differentially extend up to the ventricular surface suggests the presence of an additional factor able to modulate local concentrations of CSF factors, by directly increasing local CP secretions or by affecting CSF flow.

Geometrically, it is known that some hCP regions appear more “ordered” (i.e., the epithelium and underlying stroma form a continuous sheet or a compact 3D structure), whereas other regions appear “disordered” with long, irregularly ramified fronds. The distribution of ordered and disordered CP regions appears to be maintained in a species- and ventricle-related way (Gomez and Gordon-Potts, 1981; Weiger et al., 1986; Scala et al., 1994; Marinković et al., 2005; Zagorska-Swiezy et al., 2008) and is likely to follow gradients of morphogen expression as found in Dani et al. (2021). Although no roles have been found for these geometrical differences yet, it is interesting to note that CP volumes may vary within the human population, physiologically for some regions, such as Bochdalek’s flower basket (Horsburgh et al., 2012; Barany et al., 2017) or pathologically (with a connection to psychosis) for lateral CP (Lizano et al., 2019). In humans, the glomus of the lateral ventricles CP contains extremely tortuous capillary beds (Marinković et al., 2005) (defined “chaotic” in Zagorska-Swiezy et al., 2008), strikingly differing from the rest of CP. It would be interesting to see whether a similar organization is found as the one presented here.

Changes in the growth or positioning of different CP regions and fronds would modulate the exchange of signals with the periventricular brain tissue. CP contacts with brain parenchyma are characterized by exchange of vesicles (Terr and Edgerton, 1985) similar to those released through apocrine secretion by CP epithelium (Balusu et al., 2016); moreover, macrophages are found to exit the CP at “hotspots” at the distal end of fronds upon CCL2 signaling (Cui et al., 2020). This suggests that these contacts are not a product of chance. Furthermore, the structures where we observed CP contacts overlap with those in the 4th ventricle that display adult neurogenesis (Tighilet et al., 2007, 2016; González-González et al., 2017), suggesting a role for the hCP in neurogenesis and plasticity, as is known for the lateral ventricle CP (Kaiser and Bryja, 2020), and as would be suggested by the expression of Otx2 in both lateral and hCP (Spatazza et al., 2013). At present, mechanisms responsible for contact formation are not clear, and may involve CSF flow [which creates “currents” (Faubel et al., 2016) that may direct the flexible fronds to precise spots], developmental patterns where certain fronds are induced to grow longer by intrinsic signals and/or chemoattractants (Nielsen and Dymecki, 2010; Cui et al., 2020; Dani et al., 2021), and complementary adhesion proteins on the ependyma and CP epithelium (see for example, Lobas et al., 2012) that may induce adhesion of the CP epithelium of different villi (thereby keeping them compact) or anchor CP villi to ventricular surface spots. Developmental and molecular studies on these contacts will be needed to help clarify the issue. The present observations on microvascular pattern suggest a mechanism whereby local adult angiogenesis would modulate the structure of villi and fronds, by differentially lengthening of microvascular network compartments, and may therefore also regulate CP relations with the ventricular surface. In this context, although more observation will be needed, the preliminarily observed gender difference in number and type of contacts could underlie some of the gender-related functional CP differences, such as TTR effects (Vancamp et al., 2019).

Several clearing techniques are known to induce size changes. In particular, iDISCO+ reduces mouse brain size about 10% (Renier et al., 2016) but does not affect the size of bones (Wang et al., 2019). Although such limited shrinking does not cause bulk damage, and therefore vascular connectivity should not be distorted, several of our measured parameters are likely to be affected. In particular, connecting structures between brain regions (such as CP fronds) will be stretched, and weak connection could detach. The finding that, despite this stretch, connections are still consistently observed between CP and ventricle floor, suggests that at contact points there may be mechanisms for cell adhesion, and not simple juxtaposition of CP and ependyma; our technique would actually lead us to an underestimation of contact points. On the other hand, CP volume shrinking will certainly bias densities (e.g., of macrophages) toward slightly higher values, and would also affect local curvature of fronds and capillaries, therefore biasing tortuosity measures. Therefore, comparisons of measurements taken with iDISCO+ and vascular cast reconstruction (e.g., Quintana et al., 2019) must take this factor into account.

## Data Availability Statement

The raw data supporting the conclusions of this article will be made available by the authors, without undue reservation.

## Ethics Statement

The animal study was reviewed and approved by the OPBA – University of Pavia and Ministry of Public Health, Rome, Italy.

## Author Contributions

PP ideated the experiments, performed the surgeries, clearing, and image analysis, wrote the manuscript, and made figures. RR, CR, DC, SL, and RP performed the clearing, image analysis, and made videos. PB, FV, FH, LB, and IG performed the light-sheet scans. All authors contributed to discussion, revised and approved the article.

## Conflict of Interest

The authors declare that the research was conducted in the absence of any commercial or financial relationships that could be construed as a potential conflict of interest.

## Publisher’s Note

All claims expressed in this article are solely those of the authors and do not necessarily represent those of their affiliated organizations, or those of the publisher, the editors and the reviewers. Any product that may be evaluated in this article, or claim that may be made by its manufacturer, is not guaranteed or endorsed by the publisher.

## Footnotes

1
http://mesospim.org/

2
https://github.com/mesoSPIM/mesoSPIM-control

## Supplementary Material

Supplementary Figure 1 **(A)** Schema of the CP as in Figure 1A, with indications of arterial points of entry. Symbols and colors indicate samples. **(B)** Schema of the CP as in Figure 1A, with indications of venous points of entry. Symbols and colors indicate samples.

Supplementary Video 1 Z-stack progression of light-sheet 3D dataset of the whole-mount brain-bone preparation of Figure 2A, showing the extent and association of the CP (highlighted in red). Scale bar: 2 mm.

Supplementary Video 2 **(A)** 360° rotation on *y*-axis of reconstruction shown in Figure 8A1. **(B)** 360° rotation on *y*-axis of reconstruction shown in Figure 8A2. **(C)** 360° rotation on *y*-axis of reconstruction shown in Figure 8A3. **(D)** 360° rotation on *y*-axis of reconstruction shown in Figure 8A4.

Supplementary Video 3 Z-stack progression of light-sheet 3D dataset of the region shown in Figure 9B. Scale bar: 200 μm.

## References

[B1] Ahrens J. Geveci B. Law C. (2005). *ParaView: An End-User Tool for Large Data Visualization*, Visualization Handbook. Amsterdam: Elsevier.

[B2] Arganda-Carreras I. Fernández-González R. Muñoz-Barrutia A. Ortiz-De-Solorzano C. (2010). 3D reconstruction of histological sections: Application to mammary gland tissue. *Microsc. Res. Tech.* 73 1019–1029. 10.1002/jemt.20829 20232465

[B3] Ayachit U. (2015). *The ParaView Guide: A Parallel Visualization Application.* New York, NY: Kitware.

[B4] Balusu S. Van Wonterghem E. De Rycke R. Raemdonck K. Stremersch S. Gevaert K. (2016). Identification of a novel mechanism of blood-brain communication during peripheral inflammation via choroid plexus-derived extracellular vesicles. *EMBO Mol. Med.* 8 1162–1183. 10.15252/emmm.201606271 27596437 PMC5048366

[B5] Barany L. Baksa G. Patonay L. Ganslandt O. Buchfelder M. Kurucz P. (2017). Morphometry and microsurgical anatomy of Bochdalek’s flower basket and the related structures of the cerebellopontine angle. *Acta Neurochir.* 159 1539–1545. 10.1007/s00701-017-3234-9 28584917

[B6] Barrière D. A. Magalhães R. Novais A. Marques P. Selingue E. Geffroy F. (2019). The SIGMA rat brain templates and atlases for multimodal MRI data analysis and visualization. *Nat. Commun.* 10:5699. 10.1038/s41467-019-13575-7 31836716 PMC6911097

[B7] Cserr H. F. (1971). Physiology of the choroid plexus. *Physiological. Rev.* 51 273–311. 10.1152/physrev.1971.51.2.273 4930496

[B8] Cui J. Frederick B. Shipley M. L. Shannon O. A. Neil D. Mya D. (2020). Inflammation of the Embryonic Choroid Plexus Barrier following Maternal Immune Activation. *Dev. Cell* 2020:55. 10.1016/j.devcel.2020.09.020 33038331 PMC7725967

[B9] Dani N. Herbst R. H. McCabe C. Green G. S. Kaiser K. Head J. P. (2021). A cellular and spatial map of the choroid plexus across brain ventricles and ages. *Cell* 184 3056.e–3074.e. 10.1016/j.cell.2021.04.003 33932339 PMC8214809

[B10] Farago A. F. Awatramani R. B. Dymecki S. M. (2006). Assembly of the brainstem cochlear nuclear complex is revealed by intersectional and subtractive genetic fate maps. *Neuron* 50 205–218. 10.1016/j.neuron.2006.03.014 16630833

[B11] Faubel R. Westendorf C. Bodenschatz E. Eichele G. (2016). Cilia-based flow network in the brain ventricles. *Science* 353 176–178. 10.1126/science.aae0450 27387952

[B12] Fehrenbach J. Weiss P. Lorenzo C. (2012). Variational algorithms to remove stationary noise: applications to microscopy imaging. *IEEE Trans. Image Proc.* 21 4420–4430. 10.1109/TIP.2012.2206037 22752131

[B13] Ghersi-Egea J. F. Strazielle N. Catala M. Silva-Vargas V. Doetsch F. Engelhardt B. (2018). Molecular anatomy and functions of the choroidal blood-cerebrospinal fluid barrier in health and disease. *Acta Neuropathol.* 135 337–361. 10.1007/s00401-018-1807-1 29368213

[B14] Gomez D. G. Gordon-Potts D. (1981). The lateral, third, and fourth ventricle choroid plexus of the dog: a structural and ultrastructural study. *Ann. Neurol.* 10 333–340. 10.1002/ana.410100404 7316486

[B15] González-González M. Gómez-González G. Becerra-González M. (2017). Identification of novel cellular clusters define a specialized area in the cerebellar periventricular zone. *Sci. Rep.* 7:40768. 10.1038/srep40768 28106069 PMC5247769

[B16] Horsburgh A. Kirollos R. W. Massoud T. F. (2012). Bochdalek’s flower basket: applied neuroimaging morphometry and variants of choroid plexus in the cerebellopontine angles. *Neuroradiology* 54 1341–1346. 10.1007/s00234-012-1065-1 22777194

[B17] Huang X. Ketova T. Fleming J. T. Wang H. Dey S. K. Litingtung Y. (2009). Sonic hedgehog signaling regulates a novel epithelial progenitor domain of the hindbrain choroid plexus. *Development* 136 2535–2543. 10.1242/dev.033795 19570847 PMC2709062

[B18] Hubert V. Chauveau F. Dumot C. Ong E. Berner L. P. Canet-Soulas E. (2019). Clinical Imaging of Choroid Plexus in Health and in Brain Disorders: A Mini-Review. *Front. Mole. Neurosci.* 12:34. 10.3389/fnmol.2019.00034 30809124 PMC6379459

[B19] Kaiser K. Bryja V. (2020). Choroid Plexus: The Orchestrator of Long-Range Signalling Within the CNS. *Int. J. Mol. Sci.* 21:4760. 10.3390/ijms21134760 32635478 PMC7369786

[B20] Kirst C. Skriabine S. Vieites-Prado A. Topilko T. Bertin P. Gerschenfeld G. (2020). Mapping the Fine-Scale Organization and Plasticity of the Brain Vasculature. *Cell* 180 780.e–795.e. 10.1016/j.cell.2020.01.028 32059781

[B21] Kreeger P. K. Strong L. E. Masters K. S. (2018). Engineering Approaches to Study Cellular Decision Making. *Annu. Rev. Biomed. Eng.* 20 49–72. 10.1146/annurev-bioeng-062117-121011 29328778 PMC6327838

[B22] Lizano P. Lutz O. Ling G. Lee A. M. Eum S. Bishop J. R. (2019). Association of Choroid Plexus Enlargement With Cognitive, Inflammatory, and Structural Phenotypes Across the Psychosis Spectrum. *Am. J. Psychiatry* 176 564–572. 10.1176/appi.ajp.2019.18070825 31164007 PMC6676480

[B23] Lobas M. A. Helsper L. Vernon C. G. Schreiner D. Zhang Y. Holtzman M. J. (2012). Molecular heterogeneity in the choroid plexus epithelium: the 22-member γ-protocadherin family is differentially expressed, apically localized, and implicated in CSF regulation. *J. Neurochem.* 120 913–927. 10.1111/j.1471-4159.2011.07587.x 22092001 PMC3296866

[B24] Lun M. Monuki E. Lehtinen M. (2015). Development and functions of the choroid plexus–cerebrospinal fluid system. *Nat. Rev. Neurosci.* 16 445–457. 10.1038/nrn3921 26174708 PMC4629451

[B25] Lun M. P. Johnson M. B. Broadbelt K. G. Watanabe M. Kang Y. J. Chau K. F. (2015). Spatially heterogeneous choroid plexus transcriptomes encode positional identity and contribute to regional CSF production. *J. Neurosci.* 35 4903–4916. 10.1523/JNEUROSCI.3081-14.2015 25810521 PMC4389594

[B26] Maharaj A. S. Walshe T. E. Saint-Geniez M. Venkatesha S. Maldonado A. E. Himes N. C. (2008). VEGF and TGF-beta are required for the maintenance of the choroid plexus and ependyma. *J. Exp. Med.* 205 491–501. 10.1084/jem.20072041 18268040 PMC2271023

[B27] Marinković S. Gibo H. Filipović B. Dulejić V. Piscević I. (2005). Microanatomy of the subependymal arteries of the lateral ventricle. *Surg. Neurol.* 63 451–458. 10.1016/j.surneu.2004.06.013 15883071

[B28] Meunier A. Sawamoto K. Spassky N. (2013). “Chapter 42 Ependyma Choroid,” in *Patterning and Cell Type Specification in the Developing CNS and PNS*, eds John L. R. Rubenstein P. R. (New York, NY: Academic Press), 819–833. 10.1016/B978-0-12-397265-1.00086-1

[B29] Neman J. Chen T. (eds) (2016). *The Choroid Plexus and Cerebrospinal Fluid.* New York, NY: Academic Press, 10.1016/C2014-0-00275-4

[B30] Nielsen C. M. Dymecki S. M. (2010). Sonic hedgehog is required for vascular outgrowth in the hindbrain choroid plexus. *Dev. Biol.* 340 430–437. 20123094 10.1016/j.ydbio.2010.01.032 PMC2897143

[B31] Panezai S. K. Luo Y. Vibulyaseck S. Sarpong G. A. Nguyen-Minh V. T. Nedelescu H. (2020). Reorganization of longitudinal compartments in the laterally protruding paraflocculus of the postnatal mouse cerebellum. *J. Comp. Neurol.* 528 1725–1741. 10.1002/cne.24849 31891184

[B32] Parab S. Quick R. E. Matsuoka R. L. (2021). Endothelial cell-type-specific molecular requirements for angiogenesis drive fenestrated vessel development in the brain. *Elife* 10:e64295. 10.7554/eLife.64295 33459592 PMC7840183

[B33] Paxinos G. Watson C. (2013) *The Rat Brain in Stereotaxic Coordinates*, 7th Edn. Cambridge, MA: Academic Press.

[B34] Perin P. Voigt F. F. Bethge P. Helmchen F. Pizzala R. (2019). iDISCO+ for the Study of Neuroimmune Architecture of the Rat Auditory Brainstem. *Front. Neuroanat.* 13:15. 10.3389/fnana.2019.00015 30814937 PMC6381022

[B35] Quintana D. D. Lewis S. E. Anantula Y. Garcia J. A. Sarkar S. N. Cavendish J. Z. (2019). The cerebral angiome: High resolution MicroCT imaging of the whole brain cerebrovasculature in female and male mice. *NeuroImage* 2019 202. 10.1016/j.neuroimage.2019.116109 31446129 PMC6942880

[B36] Renier N. Adams E. L. Kirst C. Wu Z. Azevedo R. Kohl J. (2016). Mapping of Brain Activity by Automated Volume Analysis of Immediate Early Genes. *Cell* 165 1789–1802. 10.1016/j.cell.2016.05.007 27238021 PMC4912438

[B37] Sangiorgi S. Picano M. Manelli A. Peron S. Tomei G. Raspanti M. (2003). The microvasculature of the lateral choroid plexus in the rat: a scanning electron microscopy study of vascular corrosion casts. *Eur. J. Morphol.* 41 155–160. 16229157

[B38] Scala G. Mirabella N. Paino G. Pelagalli G. V. (1994). Sur la microvascularisation des plexus choroïdes des ventricules latéraux chez la Chèvre (Capra hircus) [The microvascularization of the choroid plexus of the lateral ventricles in the goat (Capra hircus)]. *Anat. Histol. Embryol.* 23 93–101. 7978353 10.1111/j.1439-0264.1994.tb00241.x

[B39] Schindelin J. Arganda-Carreras I. Frise E. Kaynig V. Longair M. Pietzsch T. (2012). Fiji: an open-source platform for biological-image analysis. *Nat.* M*ethods* 9 676–682. 10.1038/nmeth.2019 22743772 PMC3855844

[B40] Sharifi M. Ciołkowski M. Krajewski P. Ciszek B. (2005). The choroid plexus of the fourth ventricle and its arteries. *Folia Morphol.* 64 194–198. 16228955

[B41] Shipley F. B. Dani N. Xu H. Deister C. Cui J. Head J. P. (2020). Tracking Calcium Dynamics and Immune Surveillance at the Choroid Plexus Blood-Cerebrospinal Fluid Interface. *Neuron* 108 623.e–639.e. 10.1016/j.neuron.2020.08.024 32961128 PMC7847245

[B42] Spatazza J. Lee H. H. Di Nardo A. A. Tibaldi L. Joliot A. Hensch T. K. (2013). Choroid-plexus-derived Otx2 homeoprotein constrains adult cortical plasticity. *Cell Rep.* 3 1815–1823. 10.1016/j.celrep.2013.05.014 23770240 PMC4119931

[B43] Sun S. Q. Hashimoto P. H. (1991). Venous microvasculature of the pineal body and choroid plexus in the rat. *J. Electron. Microsc.* 40 29–33. 1865156

[B44] Terr L. I. Edgerton B. J. (1985). Physical effects of the choroid plexus on the cochlear nuclei in man. *Acta Otolaryngol.* 100 210–217. 10.3109/00016488509104783 4061072

[B45] Tighilet B. Brezun J. M. Sylvie G. D. Gaubert C. Lacour M. (2007). New neurons in the vestibular nuclei complex after unilateral vestibular neurectomy in the adult cat. *Eur. J. Neurosci.* 25 47–58. 10.1111/j.1460-9568.2006.05267.x 17241266

[B46] Tighilet B. Dutheil S. Siponen M. I. Noreña A. J. (2016). Reactive Neurogenesis and Down-Regulation of the Potassium-Chloride Cotransporter KCC2 in the Cochlear Nuclei after Cochlear Deafferentation. *Front. Pharmacol.* 7:281. 10.3389/fphar.2016.00281 27630564 PMC5005331

[B47] Todorov M. I. Paetzold J. C. Schoppe O. Tetteh G. Shit S. Efremov V. (2020). Machine learning analysis of whole mouse brain vasculature. *Nat. Methods* 17 442–449. 10.1038/s41592-020-0792-1 32161395 PMC7591801

[B48] Tomer R. Ye L. Hsueh B. Deisseroth K. (2014). Advanced CLARITY for rapid and high-resolution imaging of intact tissues. *Nat. Protoc.* 9 1682–1697. 10.1038/nprot.2014.123 24945384 PMC4096681

[B49] Urabe N. Naito I. Saito K. Yonezawa T. Sado Y. Yoshioka H. (2002). Basement membrane type IV collagen molecules in the choroid plexus, pia mater and capillaries in the mouse brain. *Arch. Histol. Cytol.* 65 133–143. 10.1679/aohc.65.133 12164337

[B50] Vancamp P. Gothié J. D. Luongo C. Sébillot A. Le Blay K. Butruille L. (2019). Gender-specific effects of transthyretin on neural stem cell fate in the subventricular zone of the adult mouse. *Sci. Rep.* 9:19689. 10.1038/s41598-019-56156-w 31873158 PMC6927974

[B51] Voigt F. F. Kirschenbaum D. Pagès P. E. S. Campbell R. A. A. Kästli R. (2019). The mesoSPIM initiative: open-source light-sheet microscopes for imaging cleared tissue. *Nat. Methods* 16 1105–1108. 10.1038/s41592-019-0554-0 31527839 PMC6824906

[B52] Wang Q. Liu K. Yang L. Wang H. Yang J. (2019). BoneClear: whole-tissue immunolabeling of the intact mouse bones for 3D imaging of neural anatomy and pathology. *Cell Res.* 29 870–872. 10.1038/s41422-019-0217-9 31444467 PMC6796898

[B53] Weiger T. Lametschwandtner A. Hodde K. C. Adam H. (1986). The angioarchitecture of the choroid plexus of the lateral ventricle of the rabbit. A scanning electron microscopic study of vascular corrosion casts. *Brain Res.* 378 285–296. 10.1016/0006-8993(86)90931-5 3730879

[B54] Yushkevich P. A. Piven J. Cody Hazlett H. Gimpel Smith R. Ho S. Gee J. C. (2006). User-guided 3D active contour segmentation of anatomical structures: Significantly improved efficiency and reliability. *Neuroimage* 31 1116–1128. 16545965 10.1016/j.neuroimage.2006.01.015

[B55] Zagorska-Swiezy K. Litwin J. A. Gorczyca J. Pitynski K. Miodonski A. J. (2008). The microvascular architecture of the choroid plexus in fetal human brain lateral ventricle: a scanning electron microscopy study of corrosion casts. *J. Anat.* 213 259–265. 10.1111/j.1469-7580.2008.00941.x 18624828 PMC2732044

