# Supplementary figures and images for: 3D Reconstruction of the Clarified Rat Hindbrain Choroid Plexus
Source: Front Cell Dev Biol. 2021 Jul 29;9:692617. doi: 10.3389/fcell.2021.692617 (PMC8359725; doi:10.3389/fcell.2021.692617)

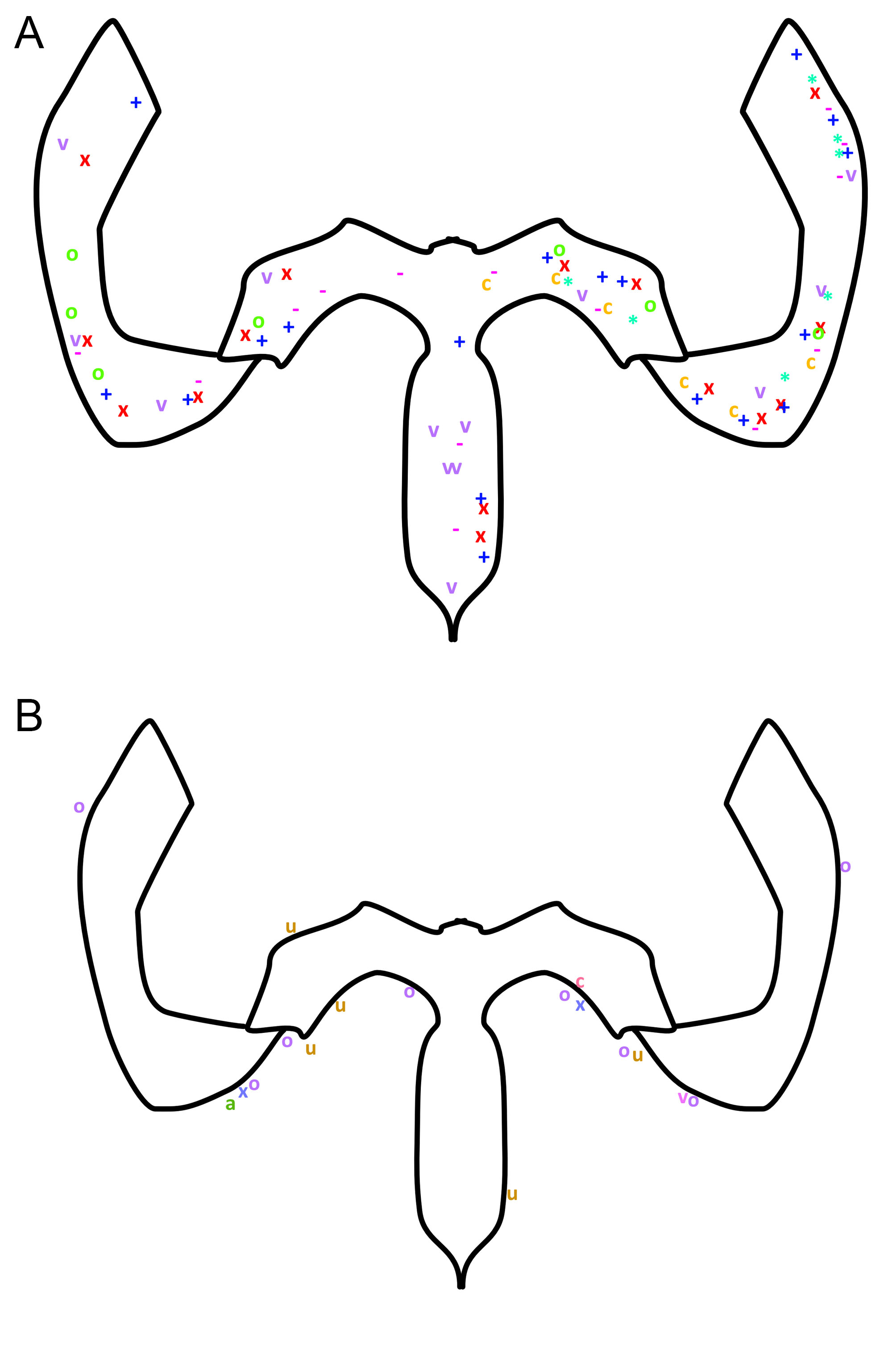

Supplement: Supplementary Figure 1 — (A) Schema of the CP as in Figure 1A, with indications of arterial points of entry. Symbols and colors indicate samples. (B) Schema of the CP as in Figure 1A, with indications of venous points of entry. Symbols and colors indicate samples. [file Image_1.jpg]
